# Borderline Clear Cell Adenofibroma of the Ovary

**DOI:** 10.1155/2017/3860107

**Published:** 2017-03-30

**Authors:** Pilaiwan Kleebkaow, Apiwat Aue-aungkul, Amornrat Temtanakitpaisan, Chumnan Kietpeerakool

**Affiliations:** Department of Obstetrics and Gynaecology, Faculty of Medicine, Khon Kaen University, Khon Kaen, Thailand

## Abstract

Borderline clear cell tumors are extremely rare, and few cases have been reported in the literature. Herein, we present a case of borderline clear cell adenofibroma of the ovary in a 58-year-old woman who presented with a pelvic mass and constipation. Physical examination revealed a 10 cm solid midline pelvic mass. Computed tomography showed an 8 cm heterogeneous enhancing mass attached to the left posterolateral wall of the uterus. The patient's serum CA 125 levels were slightly elevated (80.9 U/ml). The patient was given a total abdominal hysterectomy with bilateral salpingooophorectomy. On gross examination, it was found that the left ovarian tumor was an 8.0 × 7.5 × 8.0 cm solid multilobulated mass containing tiny cysts. Histologically, the tumor was composed of small glands in dense fibrous and myxoid stroma. The glands were lined with cuboidal cells with clear cytoplasm and mild to moderate nuclear atypia. No stromal invasion was observed. The pathological diagnosis was borderline clear cell adenofibroma of the left ovary. There was no reoccurrence 36 months post operation.

## 1. Introduction

According to the 2014 World Health Organization classification system, epithelial tumors of the ovary can be classified into three categories: benign, borderline, and malignant [1]. Histologically borderline tumors of the ovary are noninvasive epithelial tumors that show greater epithelial proliferation and cytologic atypia than benign tumors. These tumors are also referred to as tumors of low malignant potential and atypical proliferative tumors [1]. The majority of borderline tumors of the ovary are serous tumors, followed by mucinous tumors [2]. Other less common histological types include endometrioid, clear cell, and transitional cell tumors, which constitute less than 5% of all borderline tumors [2].

Borderline clear cell tumor of the ovary is defined as a tumor that is composed of glands or cysts lined by bland cuboidal to flattened cells with clear or eosinophilic cytoplasm and atypia but without stromal invasion [1]. Most clear cell tumors of the ovary are malignant. Borderline clear cell tumors are uncommon, with few case reports in the literature [1]. Therefore, an accumulation of case reports regarding these rare ovarian tumors is necessary in order to clarify their clinical behavior. Herein, we present a rare case of borderline clear cell adenofibroma of the ovary presented with a pelvic mass and constipation.

## 2. Case Report

**Figure 1:**
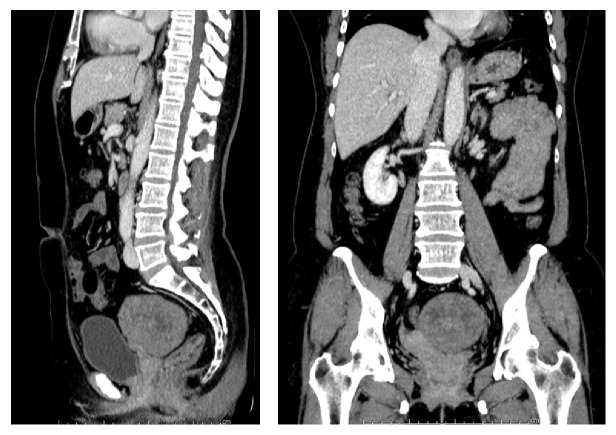
Computed tomography of abdominopelvic region shows heterogeneous enhancing mass in left side of the pelvis 8.2 × 8.0 × 7.0 cm in diameter.

A 56-year-old, G0P0 postmenopausal woman presented with a one-month history of constipation. Her past history was not remarkable. Physical examination revealed a 10 cm solid midline pelvic mass with a nodular surface. Perrectal examination revealed a firm mass compressing the anterior wall of rectum. Computed tomography (CT) of the abdominopelvic region showed an 8 cm heterogeneous enhancing mass attached to the left posterolateral wall of the uterus causing pressure effects to the rectum. Neither significant intraabdominal lymphadenopathy nor ascites were noted. The liver, pancreas, spleen, and bilateral kidneys appeared normal (Figure 1). Preoperative laboratory data indicated elevated serum CA 125 levels (80.9 U/ml). Other tumor markers such as CA 19-9 and CEA were normal. The preoperative diagnosis was subserous myoma with pressure effects.

At laparotomy, the tumor had originated from the left ovarian origin and adhered to the rectosigmoid and posterior surface of the uterus. A three-centimeter uniloculated cyst with serous content was observed in the right ovary. The uterus was macroscopically normal. There was complete obliteration of the cul-de-sac with scattered dark-brownish spots. Neither ascites nor metastasis was observed in the peritoneal cavity. A total abdominal hysterectomy with bilateral salpingooophorectomy was performed.

**Figure 2:**
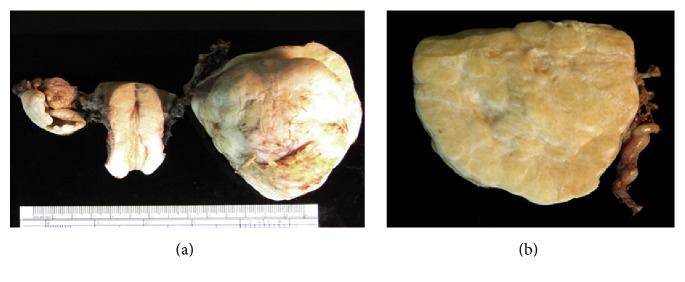
Gross examination of uterus with bilateral adnexae reveals left ovarian tumor 8.0 × 8.0 × 7.5 cm with nodular surface and a 3.0 × 3.4 × 3.4 cm thin-walled cyst at right ovary (a). Cut surfaces of left ovarian tumor show gray-white solid rubbery nodular tissues (b).

**Figure 3:**
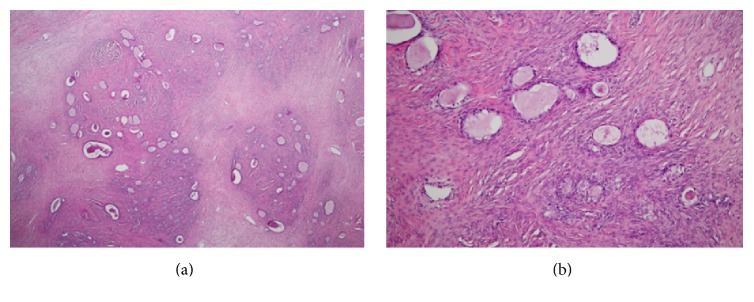
Microscopic examination reveals small glands within abundant dense fibrous and myxoid stroma (4x (a) and 10x (b)).

**Figure 4:**
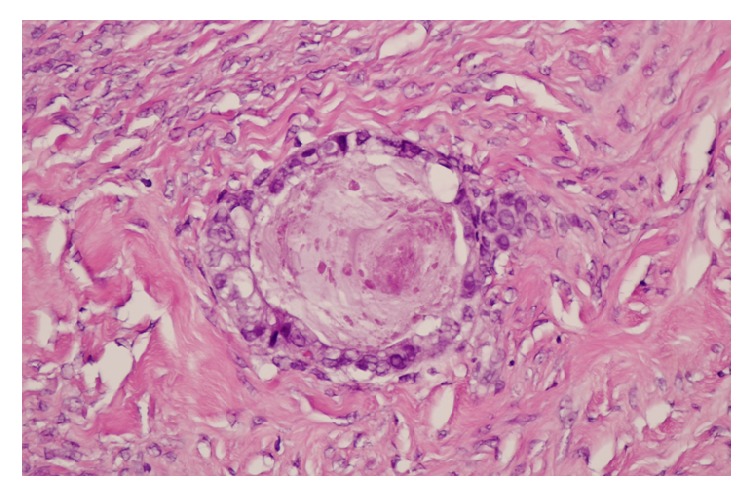
Microscopic examination shows small glands lined by bland cuboidal cells with clear cytoplasm. Mild to moderate nuclear atypia was noted. No stromal invasion was observed (40x).

Macroscopically, the left ovarian tumor was a solid 8.0 × 8.0 × 7.5 cm multinodular mass containing tiny cysts (Figure 2). Histologically, the tumor was composed of small glands in dense fibrous and myxoid stroma (Figure 3). The glands were lined with bland cuboidal cells with clear cytoplasm. Mild to moderate nuclear atypia with prominent nucleoli of the glandular epitheliums was noted. No stromal invasion was observed (Figure 4). The capsule of the left ovary was intact. Features of endometriosis were also noted adjacent to the tumor. The right ovarian cyst was a unilocular cyst lined with benign-appearing cuboidal epitheliums. The endometrium was atrophied. The uterine myometrium, cervix, and bilateral fallopian tubes were histologically unremarkable. The diagnoses of borderline clear cell adenofibroma of the left ovary and benign serous tumor of the right ovary were made based on these pathological findings. Postoperative surveillance with serial physical examination, CA-125 measurement, and CT of abdominopelvic areas were implemented. The postoperative course was uneventful with complete clinical remission for three years.

## 3. Discussion

Borderline clear cell tumors of the ovary account for less than 1% of borderline ovarian tumors [1]. Almost all patients in whom these tumors are found are postmenopausal and present with palpable masses or abdominal distension. Typically, the tumors are unilateral with solid consistency and are rarely cystic [1, 3–8]. The clinical characteristics of our patient are in line with previously reported findings.

Because of their rarity, the natural course of borderline ovarian clear cell tumors thus has yet to be fully defined. Borderline clear cell adenofibroma of the ovary has been proposed as a precursor to clear cell carcinoma, as it has a similar immunohistochemistry profile [1]. A previous study hypothesized that all types of clear cell tumors of the ovary may be derived from endometriosis along one of two possible pathways [4]. In the case of the first possible pathway, epithelial atypia arises in an endometriotic cyst and then evolves into cystic clear cell carcinoma. In the case of the second one, a fibromatous reaction occurring in an endometriotic cyst stimulates the development of clear cell adenofibroma, which subsequently progresses to borderline clear cell tumors and then to adenofibromatous clear cell carcinoma [4]. Therefore, it is not surprising that borderline clear cell tumors frequently coexist with endometriotic lesions and clear cell carcinoma on excisional specimens [4, 5, 9]. In contrast, there is an argument about whether endometriosis is a precursor lesion of adenofibromatous clear cell tumor of the ovary. A previous study from Japan noted that adenofibromatous clear cell adenocarcinoma was less frequently associated with endometriosis than cystic clear cell adenocarcinoma (14.3% versus 67.9%, resp.). This finding thus might indicate a distinct subgroup of adenofibromatous clear cell tumor from cystic clear cell tumor of the ovary [9]. In our case, the foci of endometriosis adjacent to the tumor were noted. Following thorough tissue sampling, however, no coexisting clear cell carcinoma was discovered.

Worth remembering is the fact that most borderline clear cell tumors are confined to within the ovary at presentation. Complete surgical staging and adjuvant treatment, thus, may be unwarranted in most cases. In the literature, the prognosis of borderline clear cell tumors has been favorable. Most patients remained in clinical complete remission after a follow-up period up to six years without adjuvant treatment [3–7]. In addition, complete surgical staging was not shown to be superior to incomplete staging in terms of tumor recurrence [3]. In our case, there was no extraovarian lesion detected upon preoperative investigation. The postoperative course following the total abdominal hysterectomy with bilateral salpingooophorectomy was uneventful with complete clinical remission for three years.

Interestingly, a previous study noted the relatively high rate of endometrial disorders among patients with borderline clear cell adenofibroma of the ovary [3]. Four of the 12 patients in this study had synchronous endometrial disorders. In two of these cases, this took the form of endometrial hyperplasia [3]. Although the data regarding the association between borderline ovarian clear cell tumors and endometrial disorders are limited, detailed endometrial pathology evaluation has been recommended to rule out coexisting endometrial pathology, particularly in cases of uterine preservation [3]. However, in our case, pathological examination of the endometrium revealed only findings associated with atrophic changes.

## 4. Conclusion

We presented a rare case of borderline clear cell adenofibroma of the ovary in a nulliparous, postmenopausal woman. Our report confirms the relatively good prognosis of this type of tumor. Complete surgical staging and adjuvant therapy can be omitted in most cases. However, long-term follow-ups are required to understand the biological behavior of this uncommon ovarian tumor.
